# Supplementary material for: Identifying repeat domains in large genomes
Source: Genome Biol. 2006 Jan 31;7(1):R7. doi: 10.1186/gb-2006-7-1-r7 (PMC1431705; doi:10.1186/gb-2006-7-1-r7)
Supplement: Additional File 1 — A zipped file of browsable HTML files with a complete list of the connected components in the repeat domain graph of human Repbase. [file gb-2006-7-1-r7-S1.gz › html/subgraphs/8099.pdf]

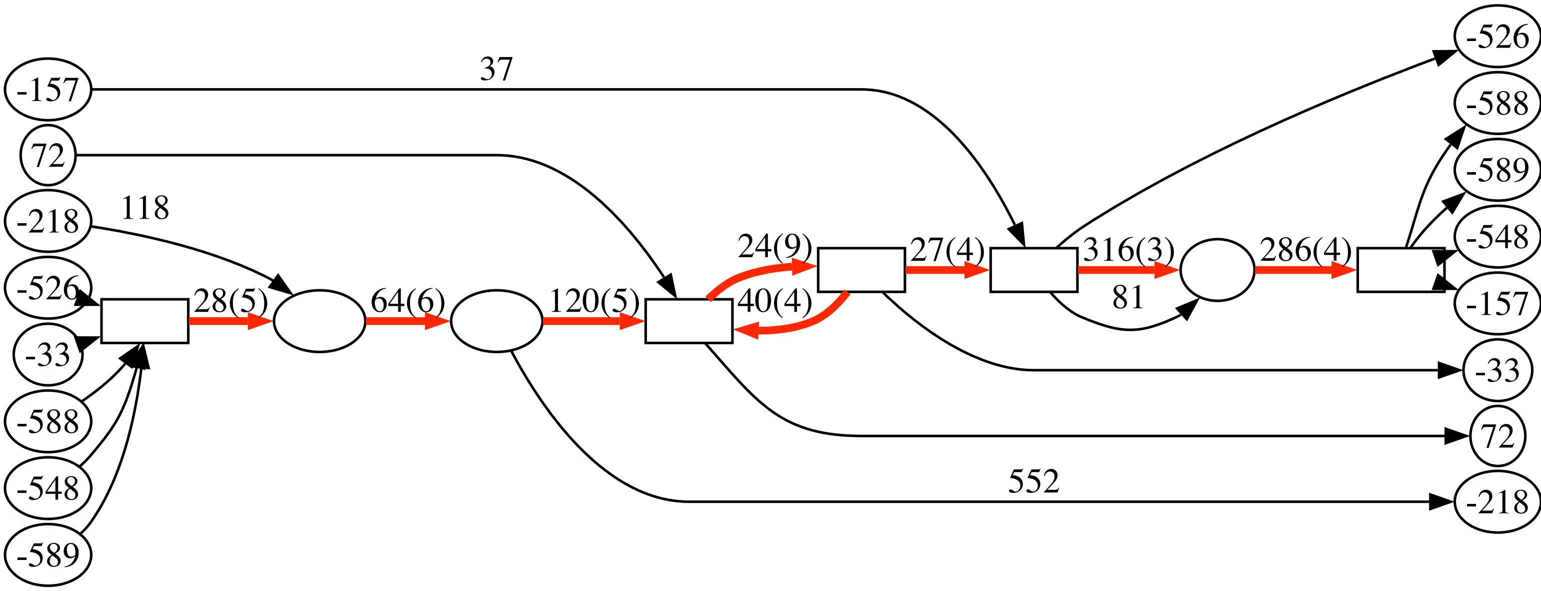

-157
37
72
-218
118
-526
-33
-588
-548
-589
28(5)
64(6)
120(5)
24(9)
40(4)
27(4)
316(3)
81
286(4)
552
-526
-588
-589
-548
-157
-33
72
-218
